# Progress in Research on Animal Collagen Peptides: Preparation, Bioactivity, and Application

**DOI:** 10.3390/molecules30153061

**Published:** 2025-07-22

**Authors:** Xuanxuan Ma, Po-Hsiang Chuang, Yu-Hui Tseng, Xiao Wang, Ziteng Ma, Haofei Chen, Wenye Zhai, Wenwen Yang, Zhaoqing Meng, Jing Xu

**Affiliations:** 1 Shandong Provincial Key Laboratory of Molecular Engineering, School of Chemistry and Chemical Engineering, Qilu University of Technology (Shandong Academy of Sciences), Jinan 250353, China; syunsma@163.com (X.M.); wangx@sdas.org (X.W.); 19063481798@163.com (Z.M.); 13970412943@163.com (H.C.); 13615470886@163.com (W.Z.); y2392015087@163.com (W.Y.); 2 College of Chemistry, Chemical Engineering and Environment, Minnan Normal University, Zhangzhou 363000, China; calcium7@hotmail.com (P.-H.C.); tsenglittleyuyu@gmail.com (Y.-H.T.); 3 Shandong Hongjitang Pharmaceutical Group Co., Ltd., Jinan 250100, China; cpummm@163.com

**Keywords:** collagen peptides, preparation, bioactivity

## Abstract

Type I collagen is a major protein in animals, and its hydrolyzed products, collagen peptides, have wide-ranging applications. This article reviews collagen peptides’ preparation methods, biological activities, and application progress in the fields of food, cosmetics, and medicine. By employing various extraction and hydrolysis methods, collagen peptides with different molecular weights can be obtained, and their biological activities are closely related to their molecular weight and amino acid sequence. Studies have revealed that collagen peptides possess a variety of biological activities, including antioxidant, hematopoietic promotion, osteogenic differentiation promotion, antihypertensive, and anti-diabetic effects. In the food industry, their antioxidant and hypoglycemic properties have opened new avenues for the development of healthy foods; in the cosmetics field, the moisturizing, anti-aging, and repair functions of collagen peptides are favored by consumers; in the medical field, collagen peptides are used in wound dressings, drug carriers, and tissue engineering scaffolds. Looking to the future, the development of green and efficient preparation technologies for collagen peptides and in-depth research into the relationship between their structure and function will be important research directions. The multifunctional properties of collagen peptides provide a broad prospect for their further application in the health industry.

## 1. Introduction

Collagen, as a biomacromolecule, is one of the most abundant proteins in the bodies of animals [1]. As early as the ancient Egyptian period, people began extracting collagen from animal skins for use as a binder [2]. In the 17th and 18th centuries, the Netherlands, the United Kingdom, France, the United States, and Germany gradually improved the industrial production techniques for collagen. In the 1950s, the triple-helix structure of the collagen molecule was first revealed. Entering the 1960s, with the advancement of biochemical technology, researchers successfully extracted collagen from animal dermal tissue and developed techniques for purifying collagen, greatly advancing the application of collagen in medical fields such as wound healing and burn treatment [3]. As time went on, research into collagen continued to deepen. In modern times, the structure, types, and biological functions of collagen have gradually been uncovered. To date, 28 types of collagens have been discovered. Common types include I, II, III, IV, V, etc. Type I collagen, consisting of two *α*1(I) chains and one *α*2(I) chain, is primarily found in skin, bones, tendons, and ligaments; it forms fibers in the dermal tissue that provide the necessary strength and support to the tissue. Type II collagen is primarily located in cartilage and the vitreous humor. Type III collagen, composed of three identical *α*1(III) chains, is widely present in the walls of blood vessels, skin, and the connective tissues of internal organs. Type IV collagen mainly exists in the basement membrane, separating and supporting cells and tissues. Type V collagen is distributed in various tissues, including skin, placenta, and blood vessels. Among all types of collagen, Type I collagen is the most widely distributed and has an extremely diverse range of uses [4]. Type I collagen is the most abundant, constituting approximately 80–90% of the total collagen in mammals, and is primarily found in skin, bones, tendons, and ligaments. Type II collagen, while less abundant overall, is the dominant collagen type in cartilage, comprising 50–80% of its dry weight, and is primarily located in cartilage and the vitreous humor [5,6]. Type III collagen, also relatively abundant (around 5–20% in skin and vascular tissues), is widely present in the walls of blood vessels, skin, and the connective tissues of internal organs [7].

In recent years, type I collagen derived from various animals has been extensively studied. Its biological activities encompass antioxidant effects, the promotion of blood coagulation, blood replenishment, anti-tumor properties, and anti-diabetic effects. Type I collagen and its hydrolyzed peptides are widely utilized in the food, cosmetics, and pharmaceutical industries. In the pharmaceutical field, type I collagen has garnered attention for its excellent tensile strength, good biodegradability, low antigenicity, low irritation, and low cytotoxicity. It is employed as a framework for artificial organs and as wound dressings to foster cell growth and adhesion. In cosmetics, collagen is primarily utilized for moisturizing, whitening, brightening the complexion, and tightening the skin to prevent wrinkles [8]. It contains natural, hydrophilic moisturizing factors that assist the skin in retaining moisture, preventing the formation of melanin, accelerating the metabolism of skin cells [9], promoting the natural shedding of the stratum corneum, eliminating pigment deposition, and reducing skin pigmentation [10]. Moreover, collagen can neutralize free radicals within the body, acting as an antioxidant, repairing broken and aged elastic fibers, thereby slowing skin aging [11]. Beyond traditional fields, collagen derivatives have recently demonstrated significant potential in environmental remediation. For instance, a modified gelatin–quaternary ammonium copolymer (MG-2) synthesized from leather collagen effectively reduced dye emissions in leather tanning processes. By adsorbing anionic dyes (e.g., >96% removal for Acid Orange II) through electrostatic interactions, this material achieved in situ wastewater treatment with residual dye concentrations as low as 23 mg·L^−1^, while simultaneously enhancing leather quality (tear strength increased by ~105%) [12]. This approach exemplifies a sustainable strategy for mitigating industrial pollution at its source. The application of natural collagen in the fields of food, pharmaceuticals, and cosmetics highlights its multifaceted functions and value, not only enriching the variety of products but also enhancing their quality and effectiveness. With the advancement of science and technology, the future application of collagen in these areas is anticipated to expand and deepen further. Recent specialized reviews have provided deeper insights into specific aspects of collagen research; for instance, advances in extraction methodologies for poultry-derived collagen and the expanding frontier of applications for marine collagen have been comprehensively documented [13,14].

This article will systematically review the preparation and bioactivity research of collagen peptides derived from various animal skins and provide a comprehensive overview of the applications of collagen peptides in health, food, pharmaceuticals, cosmetics, and other fields.

## 2. Preparation of Collagen Peptides

### 2.1. Extraction and Structural Analysis of Collagen

**Figure 1:**
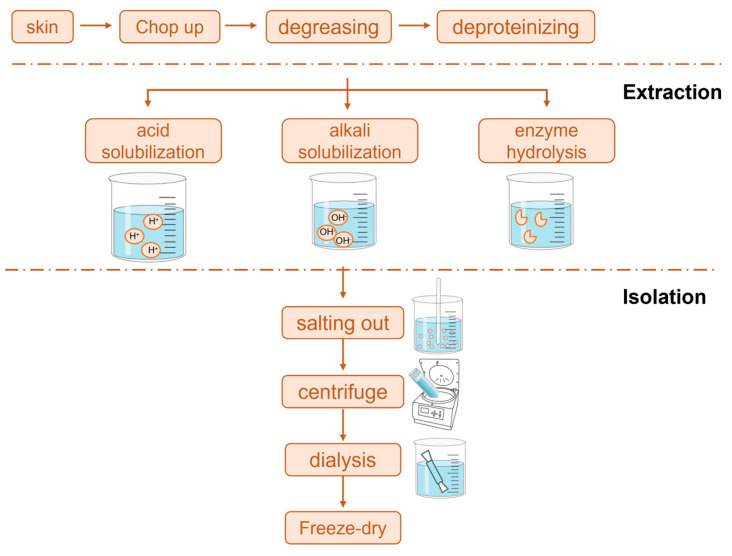
Collagen extraction methods.

Currently, the methods for extracting collagen mainly include chemical, physical, and enzymatic approaches (Figure 1). For instance, acid extraction typically uses 0.5 M acetic acid at 4 °C for 24–72 h [15,16,17,18]. Chemical methods (including the acid method [19,20,21,22] and the alkali method [23]) dominate industrial production due to their simple process and low cost. Physical methods (such as hot water extraction [24], ultrasonic assistance [25], and high-pressure homogenization [26,27]) achieve collagen extraction through gentle physical actions, but they have issues with high energy consumption and low extraction rates [28,29,30]. Enzymatic methods have become a research hotspot due to their specificity and mildness, commonly employing pepsin (0.1–1% *w*/*v*) in an acidic medium (pH 2.5–3.0) at 4–30 °C for 24–28 h [31,32,33]. This method selectively cuts the telopeptide regions of collagen without destroying its triple-helix structure, effectively maintaining the triple-helix structure and biological activity of collagen [34]. Current research trends focus on developing green and efficient composite extraction technologies, such as the combination of physical–enzymatic methods, the use of mild chemical reagents, and other innovative methods, while also integrating modern analytical techniques and artificial intelligence to optimize process parameters. Table 1 summarizes the collagen extraction methods.

**Figure 2:**
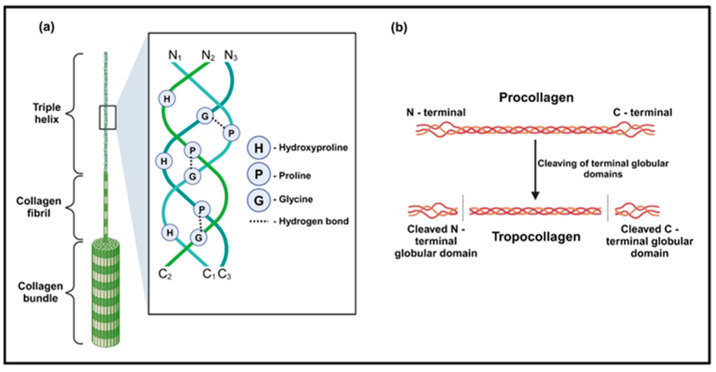
The structure of collagen. (**a**) The triple helical structure of collagen with its prominent hydroxyproline, and glycine and the hydrogen bonds formed between them (Tertiary structure). (**b**) The N-terminal and C-terminal globular domains of procollagen are cleaved to form tropocollagen [13].

After extraction, type I collagen derived from pig, bovine, chicken, fish, and sea cucumber skins exhibited certain structural differences. The main type I collagen from pigskin, extracted using an acid–enzymatic method, consists of two α1(I) chains and one α2(I) chain intertwined in a right-handed superhelical structure. The *α*-peptide chains display periodic structural characteristics, which have been extensively reported in numerous studies. It is generally accepted that each *α*-peptide chain contains approximately 1050 amino acid residues, which are periodically arranged in a Gly-X-Y sequence (Figure 2), where glycine (Gly) represents glycine, X is usually proline (Pro), and Y is usually hydroxyproline (Hyp). A study indicates that the *α*(I) chain contains 340 ± 2 repeating Gly-X-Y periodic structures, with a Gly content of about 33%, Pro content of about 23%, and Hyp content of about 10% [35]. The peptide chain exhibits a left-handed helical structure with a pitch of 0.87 nm, and each turn contains 3.6 amino acid residues [36]. The helical structure of the peptide chain is stabilized by hydrogen bonds, with the oxygen atom of each amino acid residue’s C=O forming a hydrogen bond with the hydrogen atom of the fourth amino acid residue’s N-H before it in the N-terminal to the C-terminal direction [37]. The length of the hydrogen bond (N⸱⸱⸱H⸱⸱⸱O) is approximately 2.8 Å, and the energy is about 5 kcal/mol [38]. He et al. detected *α*1(I) and *α*2(I) chains with a molecular weight of about 130 kDa using sodium dodecyl sulfate–polyacrylamide gel electrophoresis (SDS-PAGE) technology [39]. The study also showed that pigskin type I collagen exhibits a characteristic amino acid profile dominated by Gly (33%), Pro (12%), and Hyp (10%). Minor components include alanine (Ala, 11%), glutamic acid (Glu, 7%), and arginine (Arg, 5%), while cysteine (Cys) and tryptophan (Try) are present only in trace amounts (<0.5%) [40]. Importantly, this characteristic structure and amino acid profile govern not only the native collagen stability but also the solution aggregation behavior of the collagen derivative gelatin, which critically impacts its chemical reactivity, as demonstrated by concentration-dependent variations in grafting density [41].

Type I collagen extracted from bovine skin by an acetic acid–enzymatic method has a molecular weight of up to 350 kDa and consists of two α1(I) chains and one α2(I) chain intertwined in a right-handed superhelical structure. The molecular weights of the *α*1(I) and *α*2(I) chains are 135 kDa and 110 kDa, respectively [42]. Additionally, its amino acid composition is characterized by Gly (33%) and Pro (12%) as the most abundant residues. Significant levels of Ala (11%) and Hyp (9%) are observed, with other amino acids collectively constituting less than 23% of the total [43].

Collagen derived from chicken skin contains both type I and type III collagens as major components [44]. Acid-extracted type I collagen has a molecular weight of 285 kDa and comprises two α1(I) chains and one α2(I) chain intertwined in a right-handed superhelical structure. According to Gojkovic’s data on chicken skin collagen, the molecular weights of the *α*1(I) and *α*2(I) chains are 115 kDa and 130 kDa [45], respectively. Currently, specific data on the molecular weight of type III collagen has not been directly provided in the literature, and detailed information on this topic has not been reported. Chicken skin collagen is primarily composed of Gly (30%), Pro (12%), and Hyp (10%). Glu (7%), Ala (9%), and Arg (8%) are moderately abundant, whereas aromatic residues (Tyr, Phenylalanine (Phe), Trp) each constitute <3% [46].

In recent years, outbreaks of infectious diseases in terrestrial animals have limited the application of collagen derived from these sources, and religious beliefs have imposed further restrictions on the use of animal-derived collagen. These factors have spurred the development of research into marine biological collagen applications. The primary type of collagen extracted from fish skin using an acid–enzymatic method is type I, with an average molecular weight of 300 kDa. The molecular weight of the *α*1(I) chain is 116 kDa, and the *α*2(I) chain is 120 kDa [18]. Although the amino acid sequences of fish skin from different species may vary, their *α*(I) chains all contain the typical Gly-Pro-Hyp repeating sequence. Different types of fish skin contain various kinds and amounts of amino acids, but Gly, Hyp, and Pro are the main amino acids with higher content. Fish skin collagen is enriched in Gly (30–35%), Pro (10–15%), and Hyp (8–12%). Key secondary residues include Ala (11%), Glu (7%), and Arg (8%), while histidine (His), Try, and methionine (Met) are minor components (<2%) [47]. The denaturation temperature of fish skin collagen is lower than that of pig skin collagen, a fact attributed to its higher proline content and degree of hydroxylation. Hyp is crucial for maintaining the stability of the collagen trimer. The higher the Hyp, the greater the expected stability of collagen. For instance, Song et al. used differential scanning calorimetry (DSC) to determine that the thermal denaturation temperature of acid-soluble collagen (ASC) from Nile tilapia skin is 36.8 °C [48], while Menezes et al. utilized viscosity methods to determine that the thermal denaturation temperature of Nile tilapia skin collagen extracted at 4 °C is 28.9 °C [49].

The collagen fibrils of the sea cucumber Apostichopus japonicus are heterotypic [50]. Despite the established heterotypic nature of these fibrils, our current understanding of their specific *α*-chain ratios and functional mechanisms is insufficient, indicating the need for further in-depth investigation. Research has also documented the extraction of type I collagen from sea cucumbers. Amino acid and SDS-PAGE analyses of the acetic acid-pepsin extracted collagen determined the molecular weight of the α1(I) chain to be 125 kDa. Sea cucumber collagen features Gly (32%) and Ala (11%) as predominant residues, with significant Pro (9%) and Hyp (7%). Glu (10%) and Asp (6%) contribute to its acidic character, although hydrophobic residues (Val, Leu, Ile) together account for approximately 6% [51].

**Table 1:** A summary of collagen extraction methods.

Source	Extraction Method	Time (h)	Temperature (°C)	pH	Solid–Liquid Ratio (*w*/*v*)	Total Yield (%) ^a^	References
Pig skin	0.5 M acetic acid	24 h	4 °C	2.5	1:10	N/A	[15]
	0.5 M acetic acid + 0.1% pepsin	24 h	4 °C	2.5	1:10	10.8%	[31]
Cow skin	0.5 M acetic acid	24 h	21 °C	2.60	1:9	9.0%	[32]
	0.5 M acetic acid + 1% pepsin	24 h	4 °C	2.60	1:20	24.4%	[32,33]
	0.7 M acetic acid	24 h	4 °C	2.35	1:9	65.7%	
	0.5 M acetic acid + 0.1% pepsin	24 h	4 °C	2.5	1:10	7.7%	
Chicken skin	0.5 M acetic acid + 0.1% pepsin	28 h	30 °C	N/A	1:20	32.2%	[24]
Fish skin	0.5 M acetic acid	24 h	4 °C	N/A	1:10	11.7%	[16]
	0.5 M lactic acid	24 h	4 °C	N/A	1:10	11.6%	[16]
	0.5 M citric acid	24 h	4 °C	N/A	1:10	11.4%	[17]
	0.5 M acetic acid	24 h	4 °C	N/A	N/A	97.7% ^b^	
	0.5 M acetic acid	72 h	4 °C	N/A	1:40	27.7%	[18]
	alkali protease	6 h	50 °C	10	1:24	94.8% ^b^	[52]

^a^ Yield values rounded to one decimal place. Original data from references reported higher precision due to methodology. ^b^ Data calculated based on skin dry weight. N/A indicates data is either unobtainable.

### 2.2. Collagen Hydrolysis

**Figure 3:**
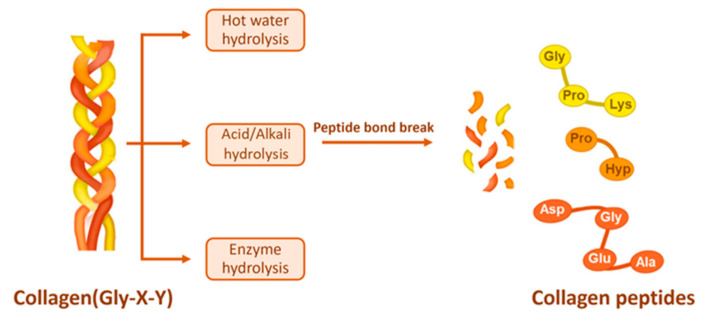
Collagen hydrolysis [59].

To obtain collagen peptides, the extracted collagen is hydrolyzed. The technology for collagen hydrolysis primarily consists of three categories: physical, chemical, and enzymatic methods. Chemical hydrolysis is further subdivided into acidic and alkaline types. In contrast to the extraction method for collagen, which frequently employs organic weak acids and weak bases, the hydrolysis of collagen typically utilizes strong acids and bases, such as hydrochloric acid and sodium hydroxide. Additionally, while the enzymes used for collagen extraction are those that specifically cleave terminal peptides, such as pepsin, the enzymes used for collagen hydrolysis cleave the peptide bonds (Figure 3).

Under acidic conditions, amino acid residues of collagen, such as aspartic acid and glutamic acid, become protonated, which reduces the stability of collagen. This process increases the concentration of positive ions, promoting the swelling and dissolution of collagen fibers [53,54]. After protonation, water molecules under acidic conditions react with the peptide bonds of collagen, leading to the breakdown of peptide chains and the production of hydrolysis products, mostly active peptides with molecular weights of 3–6 kDa, which are suitable for the food industry [55]. However, strong acids may cause excessive hydrolysis of collagen, destroying some amino acids and reducing its nutritional value and functional properties. Alkaline conditions deprotonate lysine/proline residues, with a slower reaction rate, but also capable of decomposing collagen [56,57]. Although acid and alkali methods are efficient and yield stable production, strong corrosive reagents can easily damage the nutritional structure of amino acids. The enzymatic hydrolysis method, with its precise cutting of specific peptide bonds by proteases, can controllably generate low-molecular-weight peptides of 400–2000 Da under mild conditions at 50 °C, preserving activity while ensuring high yield through optimization of enzyme combinations and process parameters (pH, temperature, enzyme dosage). Hu et al. found that adding ginger protease significantly increased the degree of hydrolysis of fish skin collagen [58]. Specifically, after the addition of ginger protease, the content of peptides with molecular weights less than 400 Da increased from 49.82% to 58.56% [58]. Wang et al. [59] compared the hydrolysis effects of pyrolysis and enzymatic hydrolysis on tilapia skin collagen, finding that enzymatic hydrolysis reduced reaction time by 70% compared to pyrolysis, required less enzyme, and offered both economic and sustainable advantages. The current technology route selection needs to balance objectives: physical methods are suitable for basic industrial scenarios, chemical methods are gradually being phased out due to environmental protection restrictions, and enzymatic hydrolysis highlights its core competitiveness in high-value-added products (such as functional foods, medical beauty materials), with its precise molecular weight control and preservation of activity becoming a key direction for industrial upgrading.

## 3. Bioactivity Analysis of Collagen Peptides

Collagen peptides exhibit biological activities including antioxidant properties, the promotion of hematopoiesis, the facilitation of osteogenic differentiation, antihypertensive effects, anti-diabetic benefits, the stimulation of hair growth, and immunomodulatory functions. These activities are closely associated with the molecular weight, the types of amino acids, and their sequences within the collagen peptides.

### 3.1. Antioxidant

Collagen peptides mitigate oxidative damage through multiple synergistic pathways, exerting antioxidant effects. The core mechanisms include the following: (1) Specific amino acid sequences in collagen peptides (such as Gly-Pro-Hyp, Pro-Hyp, etc.) can directly neutralize free radicals (such as 2,2-Diphenyl-1-picrylhydrazyl (DPPH), azino-bis (3-ethylbenzothiazoline-6-sulfonic) (ABTS), hydroxyl radicals, and superoxide anions) by providing hydrogen atoms or electrons, thereby blocking the oxidative chain reaction [60,61]. (2) Amino acids in collagen (such as histidine, cysteine) can chelate transition metal ions (such as Fe^2+^, Cu^2+^), inhibiting the Fenton reaction catalyzed by these ions that generates free radicals, reducing oxidative stress [62]. (3) Collagen peptides can activate the endogenous antioxidant system, upregulate the activity of key enzymes such as superoxide dismutase (SOD) and glutathione peroxidase (GPx), and promote antioxidant gene transcription through the Nrf2 signaling pathway, enhancing cells’ self-defense capabilities [63]. (4) Hydrogen bonding and hydrophobic interactions can reduce the reactivity of free radicals, decreasing oxidative damage to biological systems [64].

**Figure 4:**
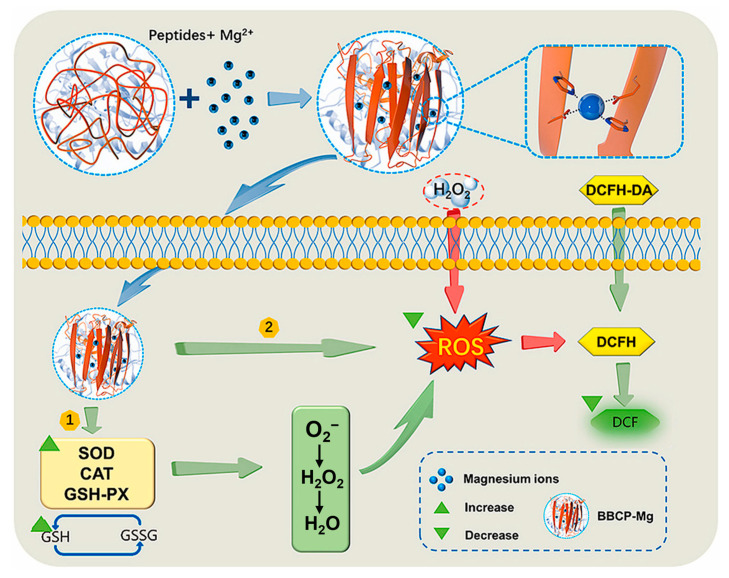
Antioxidant mechanism of peptide–magnesium chelates [62].

León-López et al. extracted sheepskin collagen using enzymatic hydrolysis and assessed its antioxidant capacity through ABTS and DPPH assays [60]. It was found that collagen peptides possess strong antioxidant activity, and the lower the molecular weight, the stronger the antioxidant capacity. This is because smaller peptides can more easily donate electrons or hydrogen atoms to stabilize free radicals [60]. Diego et al. classified collagen peptides extracted from carp by-products into four molecular weight categories: >30 kDa (PF1), 10–30 kDa (PF2), 3–10 kDa (PF3), and <1 kDa (PF4) [61]. Peptides from all molecular weight distributions exhibited antioxidant activity at different concentrations (1, 5, and 10 mg/mL). PF4 showed the highest DPPH radical scavenging activity (87%) at a concentration of 1 mg/mL and the highest hydroxyl radical scavenging activity (95%) at a concentration of 10 mg/mL, indicating that peptides with lower molecular weights have stronger antioxidant properties [61]. Zhang et al. discovered that the chelation of divalent metal ions Mg^2+^ with bovine bone collagen peptides (BBCPs) significantly enhanced the antioxidant activity of the peptides and analyzed their antioxidant mechanism [62]. Mg^2+^ chelated with BBCPs through amino nitrogen and carboxyl oxygen atoms, causing a conformational transformation in BBCPs, which altered the spatial orientation and solvent accessibility of key amino acid residues. This structural rearrangement, in turn, enhanced the scavenging activity of DPPH and ABTS radicals. BBCPs-Mg provided stronger protection and repair for oxidative damage in animal cells by inhibiting the production of ROS and increasing the activity of antioxidant enzymes (SOD, catalase (CAT), GPx) and the level of glutathione (GSH) (Figure 4) [62]. Liang et al. utilized molecular docking technology to study the interaction between the PGPAP peptide, which has the best antioxidant activity from donkey skin collagen, and the Keap1 protein [63]. The results revealed that donkey skin collagen peptides regulate the Nrf2 signaling pathway by binding to Keap1, thereby enhancing the cellular antioxidant capacity [63]. Yan et al. hydrolyzed collagen extracted from sheep hooves and used computer analysis to screen for peptides with high biological activity, ACEDAPPSAAHFR and FGFEVGPACFLG [64]. Molecular docking results indicated that both peptides have hydrogen bond interactions with DPPH and ABTS radicals, which can stabilize the structure of free radicals and reduce their reactivity, thereby decreasing oxidative damage to biological systems; the peptides also bind to free radicals through hydrophobic interactions, which may help to fix the free radicals in the hydrophobic regions of the peptides, further inhibiting their ability to participate in oxidation reactions [64]. In summary, collagen peptides have antioxidant effects and are natural antioxidants that can be developed into valuable antioxidant cosmetics or health foods.

### 3.2. Promote Hematopoiesis

Anemia is often caused by insufficient synthesis of hemoglobin or disorders in the production of red blood cells, manifesting as decreased oxygen-carrying capacity, tissue hypoxia, and metabolic disturbances [65]. The hematinic mechanism of polypeptides generally functions as a carrier for nutrients like iron and amino acids, directly participating in the synthesis of hemoglobin (for instance, polypeptides containing histidine or GSH can enhance iron absorption and heme formation) [66]; alternatively, it can activate hematopoietic regulatory pathways (such as the erythropoietin (EPO)/JAK2-STAT5 signaling pathway), thereby stimulating the proliferation and differentiation of bone marrow hematopoietic stem cells into mature red blood cells [67].

**Figure 5:**
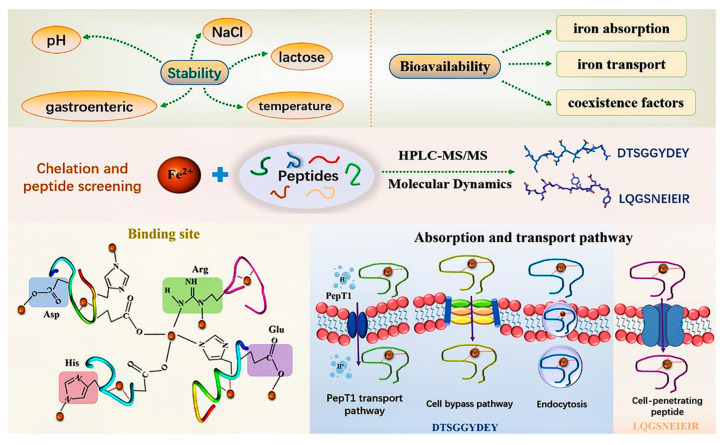
Silver carp scale collagen polypeptide iron chelation [66].

Collagen polypeptide–iron chelates can serve as iron supplements. The chelate derived from silver carp scales, known as SCSCP-Fe, is an iron supplement characterized by its excellent stability (Figure 5). This chelate achieves an iron retention rate of 81% at 85 °C, surpasses 90% in an environment with a 3.2% salt and lactose concentration, and maintains an iron retention rate of over 80% under both weak acidic and weak alkaline conditions. Following simulated gastrointestinal digestion, the iron retention rate of SCSCP-Fe remains above 75%. Experiments utilizing the Caco-2 cell monolayer model have confirmed that the bioavailability of SCSCP-Fe is significantly superior to that of the conventional iron supplement FeSO_4_ [66]. From 2007 to 2016, Wu et al. conducted research on the hematopoietic effects and mechanisms of donkey hide collagen peptides in mice with anemia induced by 5-fluorouracil, *γ*-rays, and cyclophosphamide (CTX) [67]. They found that donkey hide collagen peptides promote hematopoiesis by activating immature granulocytes and erythrocytes and partly by stimulating the granulocyte–macrophage colony-stimulating factor (GM-CSF) in all mice induced by 5-fluorouracil, *γ*-rays, or CTX. Interestingly, in mice induced by 5-fluorouracil, hematopoiesis is partly promoted through the stimulation of EPO secretion and inhibition of serum transforming growth factor (TGF-*β*) release; in mice induced by *γ*-rays, it is partly promoted through the stimulation of interleukin-6 (IL-6) secretion and the enhancement of ROS scavenging ability; and in mice induced by CTX, it is partly promoted through the stimulation of CD34 secretion and an increase in the proportion of cells in the S phase [67].

### 3.3. Promote Osteogenic Differentiation

Osteoporosis and bone defects are common pathological conditions of the skeletal system; in particular, as the population ages, the risk of fractures increases, bone function diminishes, and long-term rehabilitation becomes challenging [68]. The essence of bone formation treatment is to regulate the balance of bone metabolism (that is, to promote bone formation and inhibit bone resorption), thereby accelerating the regeneration and repair of bone tissue. The mechanism primarily involves activating osteoblast differentiation [69], inhibiting osteoclast activity [70], and enhancing the local microenvironment [71].

Research has shown that collagen polypeptides derived from starfish significantly influence the osteogenic differentiation of mouse bone marrow stem cells (BMSCs). These polypeptides drive cell differentiation towards osteogenesis by boosting the proliferation of BMSCs, increasing the activity of alkaline phosphatase, aiding the formation of cellular mineralization nodules, and upregulating the expression of mRNA for related osteogenic genes [69]. Hwang et al. explored the effects of low-molecular-weight collagen polypeptides derived from catfish skin and subjected to proteolytic hydrolysis [70]. They discovered that these polypeptides could stimulate the differentiation and mineralization of MC3T3-E1 cells in vitro and decelerate the bone remodeling process in ovariectomized rats in vivo. These results indicate that low-molecular-weight collagen polypeptides have a protective effect against bone loss in MC3T3-E1 cells and ovariectomized rats, suggesting the material can inhibit bone resorption and encourage bone formation [70]. Ding et al. synthesized highly bioactive collagen polypeptides (TBCH-a) from the bone of Thunnus orientalis using alkaline protease and found that collagen polypeptides with glutamate peptides at the C-terminus could enhance osteoblast activity [71]. They also investigated how TBCH-a induces the differentiation of MC3T3-E1 cells by activating the MAPK/ERK signaling pathway, thereby promoting the upregulation of RUNX2 at both the gene and protein levels. These findings suggest that TBCH-a can create a conducive microenvironment for the proliferation and differentiation of osteoblasts, and we believe that TBCH-a holds significant potential for improving bone healing [71].

### 3.4. Antihypertensive

Hypertension is one of the most prevalent chronic diseases globally. Long-term, uncontrolled high blood pressure markedly elevates the risk of severe complications, including cardiovascular and cerebrovascular diseases, renal failure, and more, posing a significant threat to human health and longevity. The essence of antihypertensive therapy is to maintain blood pressure homeostasis by regulating physiological processes such as vascular tone, blood volume, and cardiac function. In recent years, natural bioactive peptides have garnered considerable attention due to their high specificity and minimal side effects. The primary mechanism of action for antihypertensive peptides is the inhibition of the angiotensin-converting enzyme (ACE) [72,73].

**Figure 6:**
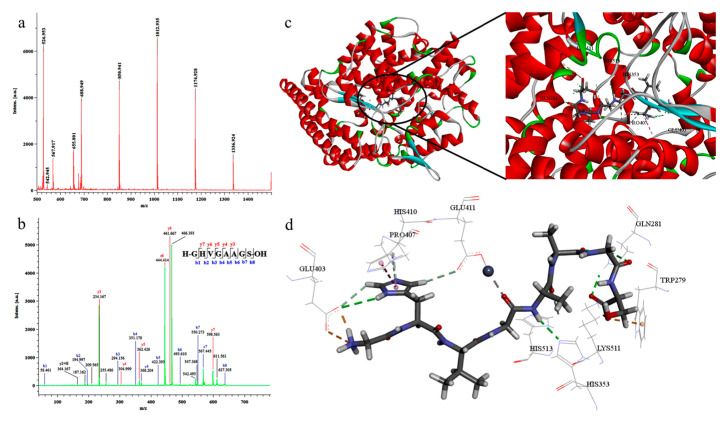
Molecular weight and amino acid sequence analysis of FCPH-IV. (**a**) LC-MS spectra of FCPH-IV. (**b**) LC-MS/MS spectra of FCPH-IV. (**c**) Putative binding mode of the peptide GHVGAAGS in the binding cavity of ACE. (**d**) Stereo view of docking poses of the peptide GHVGAAGS and the ACE molecular catalytic site. The green color represents hydrogen bonds, the orange color represents electrostatic interactions, the pink color highlights non-polar (hydrophobic) regions involved in hydrophobic contacts, and the grey color represents metal acceptor interactions [72].

Yu hydrolyzed fish gelatin and utilized ultrafiltration technology to separate four different molecular weight ranges of collagen peptides. Subsequently, peptide components with significant ACE inhibitory activity were identified. Molecular docking technology revealed the potential inhibitory mechanisms of these peptides (Figure 6). The study found that the FCPH-IV peptide segment with a molecular weight ranging from 600 to 1 kDa exhibited high ACE inhibitory activity and was identified using liquid chromatography–mass spectroscopy (LC-MS/MS) technology. A novel peptide sequence, GHVGAAGS, was identified, and it showed significant ACE inhibitory activity with an IC50 value of 407 ± 4 μM. Additionally, the in vivo antihypertensive activity of GHVGAAGS requires further study for evaluation [72]. Arby et al. [73] used thermolysin hydrolysis to break down type I collagen from cashmere goat skin and separated collagen peptides less than 5 kDa, 3–5 kDa, and less than 3 kDa in size through ultrafiltration technology. The ACE-I activity of these peptide segments was assessed using the IC50 determination method. After thermolysin hydrolysis, the molecular weight ranged from 130.81 to 17.76 kDa. The study determined the potential of thermolysin-hydrolyzed cashmere goat skin collagen to produce ACE-I peptides, where the ACE-I activity of collagen peptides less than 3 kDa ranged from 36.27 to 91.26%, with an IC50 of 82.94 μg/mL. Collagen peptides obtained through thermo-enzymatic hydrolysis of goat skin exhibited significant ACE inhibitory activity, and these peptides have potential application value in the treatment of hypertension and other areas. The study created a directed hydrolysis method, providing practical guidance for the production of functionally enhanced collagen peptides. Nonetheless, further optimization of hydrolysis conditions, identification of peptide structures, and assessments of bioavailability and safety are still required [73].

### 3.5. Anti-Diabetic

Diabetes is a metabolic disease characterized by chronic hyperglycemia. Long-term uncontrolled blood sugar can lead to serious complications such as cardiovascular disease, diabetic nephropathy, retinopathy, and neuropathy, significantly reducing the quality of life for patients and increasing the risk of mortality and disability. Collagen peptides, as a form of bioactive peptides, have shown potential application value in the field of anti-diabetes in recent years. Their mechanism of action is complex and involves multiple levels, including improving insulin resistance [74], regulating blood glucose metabolism (inhibiting the activity of *α*-glucosidase), reducing oxidative stress, and affecting the activity of related enzymes. Additionally, dipeptidyl peptidase IV (DPP-IV) is a key target for the treatment of diabetes patients. It can degrade the intestinal incretin hormones GLP-1 (glucagon-like peptide-1) and GIP (glucose-dependent insulinotropic polypeptide). By promoting insulin secretion, inhibiting glucagon release, and delaying gastric emptying, it exerts its anti-diabetic effects [33,75,76,77].

Dong et al. [74] identified a novel peptide, GPAGAP, from the skin collagen polypeptides of Andrias davidianus using network pharmacology. This peptide has been shown to improve insulin resistance in HepG2 cells and has the potential to treat type 2 diabetes mellitus (T2DM) [74]. Natsir’s enzyme kinetics analysis revealed that collagen polypeptides from the bone of Thunnus albacares exhibit competitive inhibition against *α*-glucosidase. The collagen polypeptides obtained after a hydrolysis time of 1 h demonstrated the highest *α*-glucosidase inhibitory activity, reaching 24.47% [75]. Zhou et al. employed bioinformatics methods to screen and predict the activity of type I collagen polypeptides extracted from pig skin and assessed their inhibitory effects on *α*-glucosidase in vitro [33]. Through molecular docking analysis, they identified the peptide NWYR as one of the potential core targets for treating type 2 diabetes [33]. Kusuma et al. discovered that collagen polypeptides derived from hydrolyzed Selar fish protein can enhance the expression of GLP-1 mRNA, suggesting a hypoglycemic mechanism through increased GLP-1 production [78]. They investigated the molecular docking of waste cowhide collagen polypeptides with DPP-IV targets under various preparation conditions, successfully identifying five peptides (GPVG, FGPGP, APPGAP, GPPGPT, and GPVGPPG) as potential anti-diabetic peptides, with molecular weights ranging from 329 to 596 Da [77].

### 3.6. Other Biological Activities

Kim and his research team have delved into the promotional effects of low-molecular-weight collagen peptides (LMWCPs) derived from fish on hair growth and the mechanisms of these effects. The study found that LMWCPs can accelerate the generation of new hair by increasing the secretion of growth factors and promoting the induction process of hair follicles. Furthermore, LMWCPs also enhanced the expression levels of cytokeratin as well as type I and type II keratins. In summary, LMWCPs appear promising in promoting hair growth by activating the Wnt/*β*-catenin signaling pathway [79].

**Figure 7:**
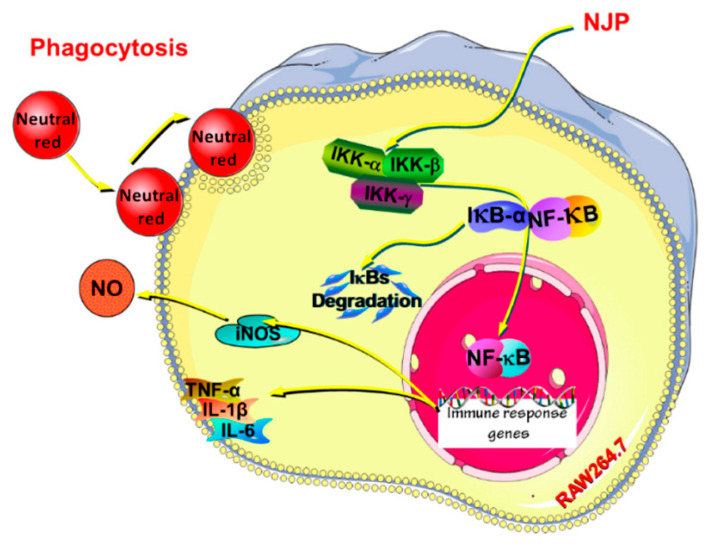
Possible mechanism of NJP exerting immunoregulatory effects in RAW264.7 cells [80]. (reproduced under terms of the CC-BY license).

Yu et al. [80] successfully isolated and purified a low-molecular-weight collagen polypeptide (MW < 3 kDa) from the hydrolysate of Nibea Japonica skin collagen (NJSP) using ultrafiltration technology. They explored the immunomodulatory effects of NJSP in a cyclophosphamide (CY)-induced immunosuppressed mouse model. By administering different doses of NJSP (100, 200, 400 mg/kg/d) via gavage, it was found that NJSP could enhance the immune organ index of mice treated with CY, repairing the damaged immune organ tissue structure. Additionally, NJSP treatment significantly promoted the proliferation of splenic cells and enhanced the phagocytic function of macrophages, while restoring delayed-type hypersensitivity. The study also showed that NJSP could significantly increase the levels of hemolysin, tumor necrosis factor *α*(TNF-*α*), immunoglobulin (Ig) A, IgG, and IgM in the serum, exhibiting dose-dependency. More notably, NJSP significantly increased the activity of SOD and CAT in the serum, enhancing the total antioxidant capacity (T-AOC) of the serum, and reducing the level of serum malondialdehyde (MDA). These findings collectively demonstrate that NJSP has the ability to protect the immune system from oxidative damage and help maintain the body’s redox balance (Figure 7) [80]. Table 2 systematically summarizes the molecular weights, main peptide segments, and corresponding bioactivities of collagen peptides from various sources.

**Table 2:** The molecular weight, peptide sequence, and corresponding effects of collagen peptides.

Sources	MW	Main Peptide Segment	Effect	References
Pork skin	<3 kDa	N/A	Antioxidant	[81]
	<1 kDa	N/A	Pancreatic lipase inhibition	[31]
	N/A	NWYR	Anti-diabetes	[33]
Cowhide	329–596 Da	GPVG/FGPGP/APGGAP/GPPGPT/GPVGPPG	Anti-diabetes	[82]
	N/A	ISVPGPM LGPVGNPGPA	ACE inhibition	[83]
Donkey hide	N/A	ACEDAPPSAAHFR/FGFEVGPACFLG	Antioxidant	[64]
		DGGR/DGD/NAGE/LVGE and GSEG	Tyrosinase inhibition	[84]
		GPAGPIGPV/GPAGPIGPV/LSSPARSGASL	Promoting hematopoiesis	[67]
Fish skin	516.5 Da	SEGPK	ACE inhibition	[85]
	597.6 Da	FDGPY		
	542.6 Da	SPGPW		
	N/A	GHVGAAGS		[72]
	N/A	SPGSSGPQGFTG/GPVGPAGNPGANGLN/PPGPTGPRGQPGNIGF	Anti-diabetes	[86]
	N/A	POGP/POGA/LPO	Contributing to fibrocyte proliferation	[87]
	358.68–863.89 Da	GPPGPPGTPGPQ/SGLPGPIGPPGPR/GLPGPIGPPGPR	Anti-photoaging	[52]
	<3 kDa	N/A	Immunoregulation	[88]
	1336 Da	FETLMPLWGNK	Anti-neurodegenerative	[89]
Sea cucumber	1336 Da	FETLMPLWGNK	Antioxidant	[90]
	1389 Da	HEPFYGNEGALR	Antioxidant Promoting wound healing	[90,91]
	961 Da	KMYPVPLN		
	N/A	VTPY/VLLY		

## 4. Applications of Collagen Peptides

### 4.1. Application in the Food Field

By utilizing the bioactivity of collagen peptides, functional foods with specific health benefits can be developed. For instance, incorporating collagen peptides into food can enhance its antioxidant capacity and delay oxidative degradation. Studies have indicated that collagen peptides inhibit *α*-glucosidase activity, suggesting their potential in the creation of hypoglycemic foods [33]. Furthermore, fish skin collagen peptides can effectively neutralize lipid peroxides, thereby protecting the lipid components in food [92]. Collagen peptides exhibit significant antioxidant properties, effectively reducing the formation of free radicals and safeguarding cells from oxidative damage [93]. Another study employing immunosuppressed mouse models demonstrated that collagen peptides contribute to increased body weight and immune-related organ indices, thus enhancing immune function [94]. Collagen also plays a beneficial role in maintaining bone density, preventing osteoporosis, and improving bone strength [95,96]. It can also enhance the immune system’s function and increase the body’s resilience. Currently, collagen primarily originates from terrestrial animals, but concerns such as the risk of disease transmission and religious restrictions persist. Consequently, the development of marine-sourced collagen has become a significant research focus. However, marine collagen exhibits lower thermal stability, which limits its use in certain food applications. Future research must focus on developing collagen products with improved thermal stability. Additionally, the extraction and production process of collagen requires further standardization to ensure the consistency and safety of product quality [97].

### 4.2. Cosmetic Applications

**Figure 8:**
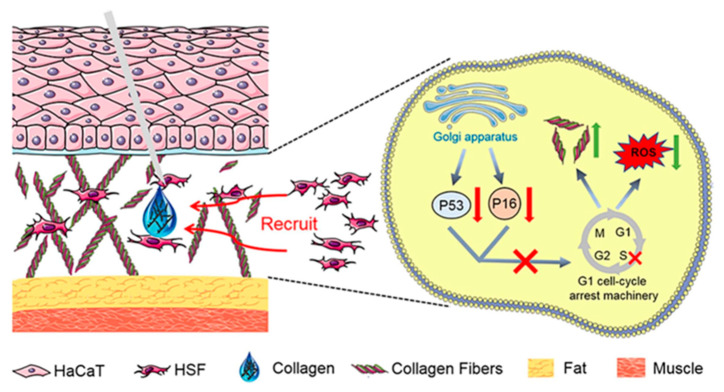
The anti-aging mechanism of collagen polypeptide for injection [15].

**Figure 9:**
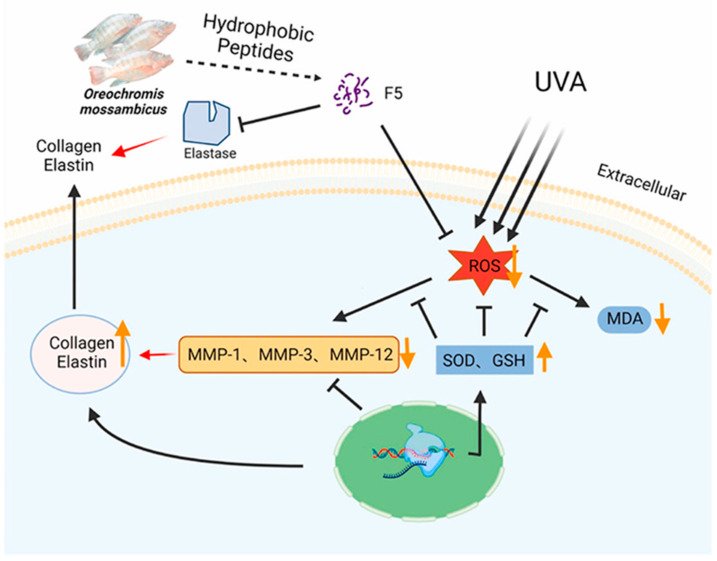
Oreochromis mossambicus-derived hydrophobic peptide F5 exhibits elastase inhibitory activity and free radical scavenging activity [99].

In the cosmetics industry, the application of collagen peptides is extremely widespread. They are renowned for their exceptional moisturizing properties, which help the skin retain moisture and alleviate dryness [98]. Collagen peptides can also promote the regeneration of skin cells, accelerate the healing process of wounds, and enhance the elasticity and firmness of the skin. In anti-aging products, collagen plays an important role as one of the main components of the skin, which is crucial for maintaining the integrity of the dermis. The loss of collagen can lead to the destruction and collapse of the dermal structure, thereby causing skin aging. Currently, the injection of exogenous collagen has become one of the effective methods for delaying skin aging. Ni He et al. extracted high-purity type I collagen from pig skin and found that it can significantly promote the adhesion and chemotaxis of human skin fibroblast (HSF) cells [15]. In addition, this collagen can reduce the expression of *β*-galactosidase, decrease the level of reactive oxygen species (ROS), and increase the expression of collagen precursors’ p53 and p16. During the aging of HSF cells, after this collagen was injected locally into the aging skin of rats, a significant increase in the number of cells and type I collagen fibers in the dermis was observed, and the arrangement of these fibers became more uniform and orderly (Figure 8) [15]. Yao et al. isolated the hydrophobic peptide F5 from collagen peptides of Oreochromis mossambicus fish scales, which exhibited multiple photoprotective mechanisms (Figure 9) [99]. F5 significantly reduced the level of ROS induced by ultraviolet A (UVA) by scavenging DPPH and hydroxyl radicals, inhibiting oxidative stress damage to fibroblasts; F5 also competitively bound to the active site of matrix metalloproteinase MMP-12 (such as Ala182 and Glu219) through molecular docking, inhibiting its elastin degradation activity, while downregulating the expression of MMP-1 and MMP-3, reducing the excessive decomposition of the extracellular matrix; in addition, F5 can promote the proliferation and migration of human foreskin fibroblasts (HFF-1) and upregulate the secretion of collagen I and elastin, thereby maintaining the integrity of the skin structure [99]. These characteristics make F5 an ideal candidate component for anti-photodamage, which can be applied to anti-aging skin care products and sunscreen formulations, providing new ideas for the diversified development of collagen resources.

To meet consumers’ demand for natural and environmentally friendly cosmetics, researchers are continually exploring new sources of collagen. Marine collagen, for example, has garnered interest because of its high bioavailability and low molecular weight, making it particularly well-suited for skincare formulations [100,101,102]. Certainly, when incorporating collagen into cosmetics, its safety and stability are of paramount importance. Studies have improved the stability and bioavailability of collagen using microencapsulation technology or chemical modification techniques, thereby ensuring its efficacy and safety in cosmetic applications [103].

### 4.3. Biomedical Applications

In the field of medicine, collagen is widely utilized in tissue engineering, wound healing, and regenerative medicine. It is employed as a sponge-like material to facilitate the healing of wounds or burns or to control hemorrhaging [80,104]. The literature indicates that fish-derived collagen is preferred for its unique physicochemical properties and can be utilized to produce cost-effective medical dressings [105]. Notably, research extends beyond simple dressings to include functionalized versions incorporating antimicrobial agents (e.g., silver nanoparticles, natural extracts) [106,107], growth factors (e.g., VEGF, EGF) [108], or stimuli-responsive components (e.g., pH-sensitive hydrogels) [109] for controlled therapeutic release.

Collagen peptides, being natural biodegradable polymers, provide significant benefits in controlling drug release and serve as a controlled material for transdermal drug delivery systems. By encapsulating drugs within a collagen matrix, they are protected from external environmental factors, such as pH fluctuations and enzymatic degradation, thereby prolonging the effective life of the drugs [110]. Collagen polypeptides can be engineered into various structures to meet different requirements, such as sponge-like, film-like, or microsphere-like, to regulate the release rate of drugs, ensuring that they are continuously released over a specific period [111]. Furthermore, collagen-based nanoparticles are actively explored for enhanced systemic or localized delivery of peptides, small molecules, and even genetic material (DNA, siRNA) [112]. In the context of transdermal drug delivery systems, collagen polypeptides assist in overcoming the barrier of the stratum corneum, facilitating easier penetration of drugs through the skin and into the bloodstream, thereby enhancing the bioavailability of drugs [113]. Additionally, because of their excellent biocompatibility and biodegradability, collagen polypeptides can decrease stimulation and toxicity reactions within the body [114].

As a macromolecule, collagen can construct structures that support cell growth and differentiation (such as cell scaffolds), which is crucial for tissue engineering. Researchers have employed a variety of techniques to prepare collagen-based scaffolds. For instance, Yang et al. used the freeze-drying method to prepare porous collagen scaffolds [115]. Additionally, studies have adopted electrospinning technology to fabricate collagen scaffolds with nanofibrous structures that mimic the microstructure of natural bone tissue, which aids in bone tissue regeneration [116]. In addition, collagen derivatives (such as gelatin) have demonstrated significant advantages in the design of functional hydrogel scaffolds. Recently, Song et al. developed a multifunctional hemostatic hydrogel based on gelatin/hyaluronic acid [117]. Through photocross-linking pretreatment performed to coordinate the hydrophilic and hydrophobic balance of the system, rapid temperature-sensitive gelation within 6–8 s, a wet adhesion strength of 125 kPa, and a burst pressure of 282 mmHg were achieved. This injectable hydrogel has an antibacterial function and promotes blood coagulation and wound healing, providing a new technical path for emergency hemostasis and tissue regeneration [117]. Significant research focuses on creating advanced composite scaffolds by blending collagen with polymers like chitosan, silk fibroin, alginate, or synthetic polymers to enhance mechanical strength and bioactivity [118]. These scaffolds are functionalized with bioactive peptides or growth factors to direct specific tissue regeneration [119]. Key application areas include engineering constructs for skin (bilayered), bone/cartilage (often mineralized), vascular (tubular grafts), nerve (aligned conduits), and corneal repair [120].

## 5. Conclusions

In the future, research on collagen will progress towards a deeper and more comprehensive direction. Developing more environmentally friendly and efficient extraction methods, such as utilizing biocatalytic enzymes and new physical methods to reduce environmental pollution and enhance the extraction efficiency and activity of collagen, will be a crucial focus. Additionally, it is essential to conduct in-depth studies on the structure–activity relationship of collagen peptides, necessitating the use of modern analytical techniques to identify active peptide segments of collagen and analyze their structures. Establishing a database of structures and biological activities will provide theoretical guidance for the development of collagen products. The development of new types of collagen products is also of significant importance, including collagen products with specific functions, such as antioxidant cosmetics, health foods that promote osteogenesis, anti-diabetic drugs, and the use of genetic engineering technology to produce recombinant collagen, as well as the development of collagen nanomaterials. It is believed that through the aforementioned research, collagen will play a more significant role in the future, contributing greatly to human health.

## Data Availability

No new data were created or analyzed in this study.
